# Immigrant life expectancy and disability-free life expectancy by detailed country of birth in England and Wales: A total population, repeated cross-sectional approach

**DOI:** 10.1371/journal.pone.0356481

**Published:** 2026-08-19

**Authors:** Joseph Harrison, Matthew Wallace

**Affiliations:** 1 School of Health and Society, University of Salford, Salford, United Kingdom; 2 Stockholm University Demography Unit (SUDA), Stockholm University, Stockholm, Sweden; Bar-Ilan University, ISRAEL

## Abstract

Research regularly reports lower mortality among migrants, leading to conclusions of a “Healthy Immigrant Effect”. However, evidence on migrant health is less consistent, suggesting a disconnect between mortality and health outcomes. This highlights the need to examine both health and mortality simultaneously rather than relying on single-outcome measures. We estimate life expectancy (LE) and disability-free life expectancy (DFLE) at age 50 for 37 countries of birth in England and Wales. Life tables were constructed using whole-population mortality data from 2000–02, 2010–12 and 2020–22, while DFLE was estimated using the Sullivan method and self-reported limiting long-term illness data from the 2001, 2011 and 2021 censuses. By comparing LE and DFLE with those of the England and Wales-born population across three time points, we assessed differences in both mortality and disability and examined how these changed over time. We find substantial heterogeneity in migrant health. Most migrant populations consistently experienced a “healthy migrant effect”, with higher LE and DFLE than the England and Wales-born population at all three time points. In contrast, several of the largest migrant groups exhibited an “unhealthy migrant effect”, characterised by lower LE and DFLE (including those born in Scotland, the Republic of Ireland, and men born in Poland and Lithuania), while others experienced longer lives but greater disability burdens, with higher LE but lower DFLE (including women born in Pakistan and Bangladesh). These findings demonstrate that migrant populations are far from homogeneous and some groups face elevated burdens of disability and/or premature mortality and would benefit from targeted public health interventions.

## Introduction

As the global population ages [1], and the share of people living in other countries rises worldwide [2], the health of immigrants has the potential to increasingly shape future population health and healthcare demands [3]. Immigration is typically seen as *the* solution to population aging, yet immigrants often “age-in-place” in their host countries and may do so in ways that differ from non-migrants [4]. This makes research into immigrant health crucial for policymakers and practitioners alike. Previous findings consistently report lower mortality risks among immigrants compared to non-migrants in high-income host countries. These lower mortality risks can persist for many decades and are strongest among immigrants born in lower, middle-income countries and young adults [5,6]. This has often been interpreted to mean that immigrants are in more robust health than non-migrants are—the “Healthy Immigrant Effect” **(HIE)** [7]. However, the evidence related to health itself is less definitive. Better health than non-migrants *is* often observed upon arrival, but these advantages quickly wear off (i.e., within 5 years) and can even reverse [8]. Systematic reviews of the evidence also report worse self-rated health among immigrants [9], combined with higher incidences of cardiovascular diseases, diabetes mellitus, respiratory diseases, and communicable diseases [10–13]. Simultaneously, evidence on dementia [14] and cancer [15] is inconclusive, with reviews reporting a large degree of heterogeneity in risks compared to non-migrants.

This highlights a clear need for more nuance in immigrant health research and to progress beyond single-outcome estimates by incorporating both immigrant health *and* mortality [16]. This echoes the general shift in focus of international health agencies, such as the *World Health Organisation,* away from traditional estimates of life expectancy **(LE)** to estimates of healthy expectancies **(HE)** that better reflect the *quality* of years people live and not merely the *quantity* [17]. **HEs** permit more accurate planning of healthcare (through anticipating demands for long-term care and the management of chronic diseases), social policy planning (in welfare, pension, and retirement systems), and targeted public health interventions for society’s most vulnerable groups [18]—which regularly include immigrants [19]. Due to stringent data demands, there are few studies of immigrant **HE** alongside **LE**. Those that *do* exist have had to rely upon smaller-scale survey data for information on health [20–23], have investigated a single point in time [24,25], analysed a single aspect of health [23,26,27], and/or have had to combine immigrants’ countries of birth into very broad origin groupings that surely mask salient differences at a more granular level [23,28,29]. Despite such challenges, these studies highlight heterogeneity in the “immigrant experience” —some groups are living longer lives in better health, while others are living longer lives in worse health [16,24,25,28,30,31].

Here, capitalizing on a repeated cross-sectional approach using comprehensive, total population data on the resident population of England & Wales, we investigate differences in **LE** and disability-free life expectancy (**DFLE**—using limiting long-term illness [LLTI]) among immigrants at three time points (2000−2, 2010−12, 2020−22) at the lowest level country of birth. We aim to provide the most up-to-date, granular estimates of **LE** and **DFLE** not only in England & Wales, but in the global migrant health evidence base so far. We identify England & Wales as a major migrant-receiving country (16.8% of the population was born outside of England & Wales in 2021 [32]) that showcases real diversity in its migration history. Immigrants in England & Wales encompass intra-UK and Irish migration, labour and family migration from (now former) colonies all over the world, recent labour migration from the European Union (EU), highly skilled, highly qualified migration from countries such as India, China and Nigeria, alongside forced migration flows, notably from the Middle East and Sub-Saharan Africa [33].

## Materials and methods

We use two sources of information. Exposure and health information is based upon “de jure” census counts, while mortality information is based on “de facto” vital registration data. Specifically, for the prevalence of LLTI and the population denominator, we use population counts from national censuses in 2001, 2011 and 2021 that were custom ordered from the Office for National Statistics (ONS) *Census Team*, and disaggregated by age (0−1, 5−9, 10−14 […], 90+), sex (men and women) and country of birth (36 lowest level countries and various aggregated regions). We also use mortality counts made available to us by the ONS *Health Data Team*. Again, this information is broken down by age, sex, and country of birth (same disaggregation as for census data). To increase the number of deaths observed, and increase statistical power, we use deaths from the census year and one year on either side. This means that we count all-cause deaths in 3-year periods in 2000−2, 2010−12 and 2020−22.

Prior to the receipt of the data, the Office for National Statistics addresses missing and inconsistent data through fixing inconsistent answers (i.e., “item editing”) and item non-response through imputation (specifically deterministic editing, nearest neighbour donor imputation and manual imputation). It also addresses under-coverage through a combination of a coverage survey (the Census Coverage Survey [CCS], dual system estimation and a final statistical coverage adjustment). These corrections have been developed and validated over successive censuses since 2001. More information about these methodologies is available online [34–36].

**Fig 1:**
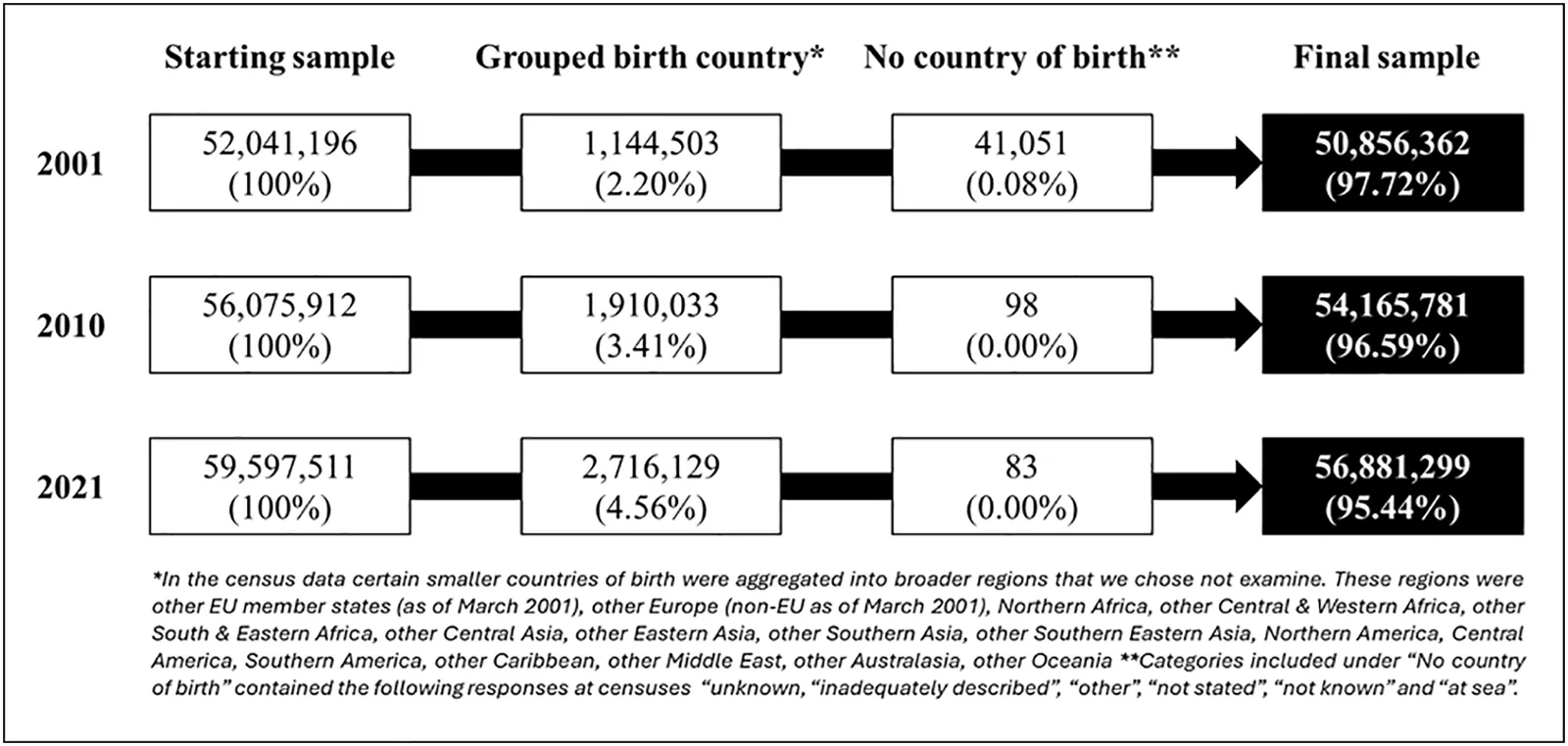
Starting sample, final sample, and exclusion criteria in 2001, 2010, and 2021. Source: Authors’ calculations based upon *Office for National Statistics* information licensed under the *Open Government Licence v.3.0*.

In **Table 1**, we report the LLTI question asked at each census, the number of individuals that answered “yes” and “no” to each question, the number of deaths in the 3-year period around each census, and the age standardised percentage in England and Wales who report having a LLTI. The starting sample, exclusion criteria, and final sample can all be found in Fig 1.

**Table 1:** Long term illness question and answers across England and Wales censuses.

Year	Question on limiting long-term illness	Answer (distribution)	LLTI age-standardised Percentage
**2001**	Do you have any long-term illness, health problem or disability which limits your daily activities or the work you can do? *(Including problems which are due to old age)*	Yes: 9,292,010 No: 41,564,352 Total: **50,856,362**	England: 19.3% Wales: 24.1%
		Deaths: **1,578,145**	
**2011**	Are your day-to-day activities limited because of a health problem or disability which has lasted, or is expected to last, at least 12 months?	Yes (limited a lot/ limited a little): 9,803,276 No: 44,362,505 Total: **54,165,781**	England: 19.3% Wales: 23.4%
		Deaths: **1,446,463**	
**2021**	Do you have any physical or mental health conditions or illnesses lasting or expected to last 12 months or more? *Subsequent question asks whether these conditions or illnesses reduce ability to carry out day to day activities?	Yes: 10,151,070 No: 46,730,299 Total: **56,881,299**	England: 17.7% Wales: 21.1%
		Deaths: **1,720,959**	

Source: *Office for National Statistics* information licensed under the *Open Government Licence v.3.0*.

Notes: **(1)** deaths cover the period 1^st^ Jan 2000–31^st^ December 2002; 1^st^ Jan 2010–31^st^ December 2012 and 1^st^ Jan 2020–31^st^ December 2022.

**(2)** lowest level distributions of population sizes, limiting long-term illness, and deaths by sex and country of birth are available in **online** **S2 File**.

**(3)** *age-standardised LLTI percentages obtained from ONS statistical bulletins (2011 and 2021) and datasets (2001)* [37,38].

To calculate the estimates for **LE** and **DFLE** we use an abridged life table (5 year age gaps, closing the life table at 90+) method and the Sullivan method [39]. We create a life table at each census point for each country of birth and sex. We use R packages ‘*Demotools*’ [40] and the Public Health England produced ‘*PHEindicatormethods*’ [41] to derive life expectancies and confidence intervals. We estimated **DFLE** using the Sullivan method, which is widely used for estimation of healthy or disability-free life expectancies [24,28,42]. For each age interval the person-years lived were multiplied by the proportion of the population reporting no disability in that age group. These disability-free person-years were summed across remaining ages and divided by the number surviving to estimate **DFLE** at each age. For the confidence intervals we performed a proportion test around the proportion non-disabled to derive upper and lower estimates of disability prevalence. Although there are well-known limitations to the Sullivan method that we acknowledge [43], we do not have sufficiently detailed longitudinal data in England & Wales to permit a robust, incidence-based approach. To maximise our comparability with previous research, we report results for life and disability-free life expectancies at age 50 (**LE50** and **DFLE50**). Estimates for expectancies from birth and age 20 are in **Online** **S1 File**.

After deriving the estimates for **LE50** and **DFLE50** for each country of birth at each time point, we compare them to the estimate of those born in England and Wales. Based on these comparisons, migrant groups were categorised according to whether **LE** and **DFLE** were higher or lower than the native-born population in 2020−22. As shown in **Table 2**, this results in four exclusive categories. For example, groups with both higher **LE** and higher **DFLE** than the England and Wales-born population were classified as exhibiting a **HIE**.

**Table 2:** Matrix of the categorisation of the relationship between life expectancy and disability-free life expectancy.

	Higher LE	Lower LE
Higher **DFLE**	Healthy Imigrant Effect (**HIE**)	Shorter Lives, Better Health (**SLBH**)
Lower **DFLE**	Morbidity-Mortality Paradox (**MMP**)	Unhealthy Imigrant Effect (**UIE**)

We choose the 2020−22 period because it represents the most recent and policy-relevant period, providing the clearest picture of the contemporary health of migrant populations. However, it is important to acknowledge that some groups have changed over time.

Our categorisation is based solely on differences in point estimates. We adopted this approach for simplicity and because the analyses are based on comprehensive population data, making the observed values substantively meaningful. We recognise, however, that alternative approaches could have been used, including consideration of the magnitude of differences, overlap in confidence intervals, or the proportion of remaining life spent disability-free. Consequently, the classification involves an element of subjectivity. Nevertheless, we believe that this framework is both useful and necessary for summarising patterns across the large number of countries included in the analysis. Confidence intervals are presented in the figures and in **Online S1 File** to allow readers to assess the uncertainty surrounding the estimates.

We performed a range of supplementary analyses. We generated estimates of healthy life expectancy (**HLE**) using self-reported health (**SRH**) derived from the same sources as described above (**Online** **S1 File**) and generated estimates for **DFLE** and **HLE** at ages 0 and 20. We additionally generated population pyramids for 2001 and 2021 to get an idea of the variation and change in population age structures among immigrant groups (**Online** **S2 File**). The same file includes population sizes by country of birth and how they have changed over time, alongside the number of reports of disability and deaths at each time point by country of birth and sex.

## Results

**Fig 2:**
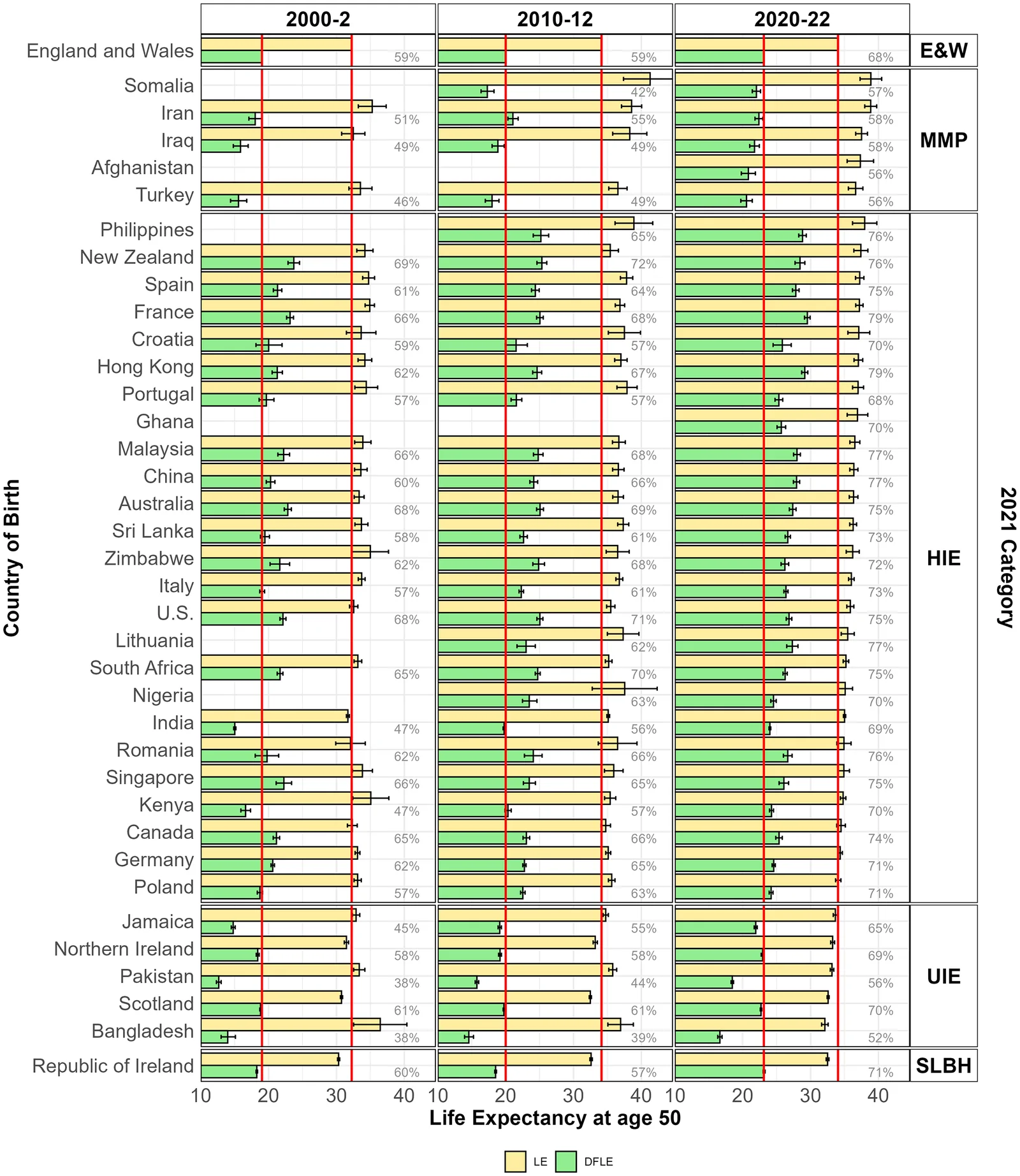
Life expectancy and disability-free life expectancy by country of birth in England and Wales for women. Notes: **(1)** Origin countries are ranked within 2020−22 typologies by life expectancy in 2021. **(2)** Percentage figure is the remaining life expected to be disability-free. Source: Authors’ calculations based upon *Office for National Statistics* information licensed under the *Open Government Licence v.3.0*.

**Fig 3:**
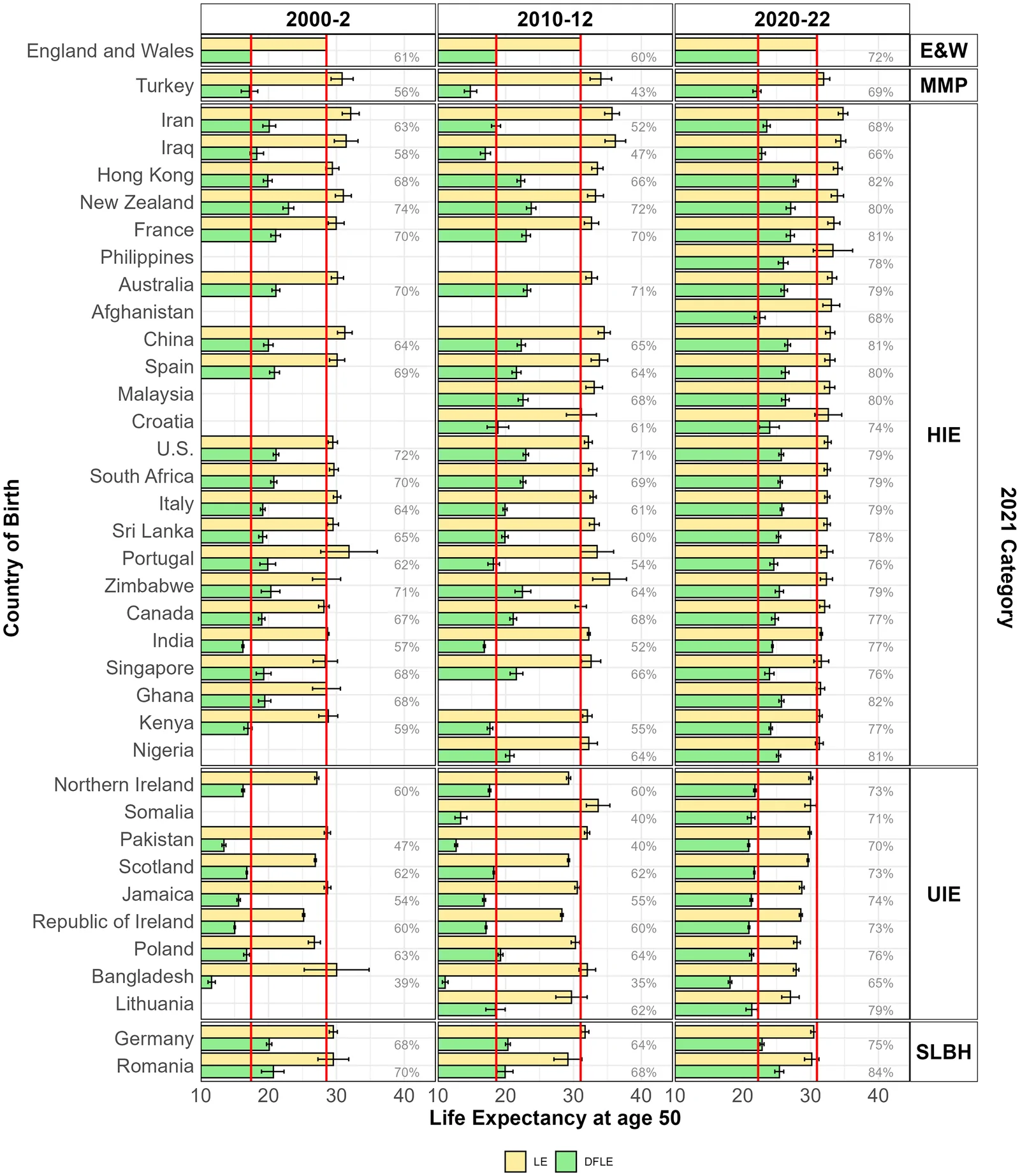
Life expectancy and Disability-Free Life Expectancy by country of birth in England and Wales for men. Notes: **(1)** Origin countries are ranked within 2020−22 typologies by life expectancy in 2021**(2)** Percentage figure is the remaining life expected to be disability-free. Source: Authors’ calculations based upon *Office for National Statistics* information licensed under the *Open Government Licence v.3.0*.

Figs 2 and 3 display the results of **DFLE** and **LE** at age 50 for women and men respectively. No estimates are shown where the confidence interval for LE exceeds 10 years, a consequence of low numbers of immigrants and/or few deaths. **Online** **S3 File** contains figures showing the same information but oriented on the differences between the point estimates for each group compared to the England and Wales-born at each time point.

For most groups of immigrants there is consistent evidence of the **HIE**, with **LE** and **DFLE** being higher, and for many groups this is the case at all three time points. Nearly all Western European, Oceanic, and North American-origin immigrant women and men consistently belong to this group. Simultaneously, there is representation from around the world, including from Sub-Saharan Africa (Ghana, Nigeria, Zimbabwe, South Africa) and Asia (Hong Kong, Singapore, Sri Lanka, Malaysia, and the Philippines). The values of **LE** and **DFLE** vary across time points, but men and women born in France, Hong Kong, New Zealand, Spain and Philippines consistently display some of the highest **LE** and **DFLE** values. Additionally, in nearly all cases of **HIE**, the % of life spent disability-free is higher compared to those born in England & Wales—though there are some exceptions. These are, for 2010−2 and 2020−2, men born in Iran and Iraq, who have a consistently lower % of life spent disability-free, a function of higher longevity and higher **LE** values, without higher **DFLE** values.

Among women, cases where both **LE** and **DFLE** are lower in 2021, an **UIE**, are found for origin groups Jamaica, Pakistan, Bangladesh, Northern Ireland and Scotland (Fig 2). The same is true for migrant men born in these countries (Fig 3). However, they are also joined by men born in Poland, Lithuania, Somalia and the Republic of Ireland (Fig 3). These findings for men born in Poland and Lithuania contrast highly with their female counterparts, who in 2021 have comparable or higher **LE** and **DFLE** compared to non-migrant women. In most cases where the **UIE** effect is found the percentage of life spent disability-free is similar to, or higher than, that of the corresponding value for non-migrant men and women born in England & Wales. A notable exception for this is Pakistani and Bangladeshi men and women who have a lower proportion of life expected to be disability-free compared to those born in England and Wales.

We observe that a handful of groups are living longer than non-migrants but spend fewer years disability-free—evidence of the **MMP**. This is found among women and men born in Turkey and women born in Somalia, Iraq, Afghanistan and Jamaica—several of which are refugee-sending countries. Moreover, several instances where the **UIE** is observed in 2021 are in groups that have shown a marked deterioration in their mortality over time and in previous censuses would have been classed as showing the **MMP.** In particular, Pakistani, Bangladeshi, and Jamaican immigrants who have had **DFLE** consistently lower than non-migrant women and men in England & Wales over time and have transitioned from having a higher **LE** than non-migrants in 2000−2 and 2010−12 to a lower **LE** by 2020−2.

Importantly, a small number of groups display an improvement in their situation over time. Women and men born in Kenya have transitioned from living longer lives with fewer years spent disability-free in 2000−2 (**MMP**) to living longer lives with more years spent disability-free in 2020−2 (**HIE**), owing to improvements in disability status. Meanwhile, women and men born in India have transitioned from living comparably long lives to non-migrant women and men in 2000−2, but with fewer years spent to disability-free, to living longer lives with more years spent disability-free (**HIE**) by 2020−2, gaining both **LE** and **DFLE**. Expectedly, these groups also make gains in their % of life that is spent disability-free.

A few groups show a unique position of shorter lives in better health (**SLBH**). In 2020−2 this was found for women born in the Republic of Ireland. They had transitioned from having **LE** and **DFLE** consistently lower than the majority population in the first two time points to **DFLE** becoming marginally above in 2020−2. Amongst men **SLBH** can be seen for Germany and Romania. Here **LE** has remained similar or below that of the England and Wales-born whilst having higher **DFLE**, as a result Romanian men have the highest proportion of life spent disability-free from any group studied at 85% in 2020−22.

### Sensitivity analyses

From **Online** **S1 File**, we report that healthy life expectancy (**HLE**) is typically 4–6 years higher among men and 6–8 years higher among women. The patterns are consistent with **DFLE** across countries of birth. All **HIE** groups that have higher **LE** and **DFLE** also exhibit a higher **HLE** than non-migrants. Similarly, **MMP** groups that have higher **LE** and lower **DFLE** also have lower **HLE** and/or lower % of years spent in good health than non-migrants do. This is unsurprising given how much self-rated health and limiting long-term illness are correlated [44]. Simultaneously, **SRH** and **LLTI** *do* capture different aspects of health. **LLTI** provides a more objective, function-focused measure of (chronic) morbidity and daily limitations, while **SRH** provides a more subjective and holistic view of wider health (including issues that are yet to be diagnosed), its perceptions, and determinants [45]. **Online** **S1 File** reinforces our findings, adds valuable additional evidence based upon a complementary measure of health, and shows that nearly all instances of **HIE**, **UIE** and **MMP** can be extended to broader definitions of health. Additionally, aside from increases in the values of **LE** and **DFLE** themselves, group membership remains consistent by birth country for **LE0**, **LE20**, **DFLE0** and **DFLE20**.

### Supplementary analyses

**Online** **S2 File** displays population pyramids, changes in age structures, and population growth within our immigrant groups between 2001 and 2021. It tells us that while nearly all immigrant groups were young in 2001 and remained so in 2021—indicating continual inflows of young adult migrants (India stands out as a notable example), several groups grew older and/or contracted in size (e.g., Northern Ireland, Scotland, the Republic of Ireland, Germany, Kenya, and Jamaica)—indicating the end of these particular inflows. As of 2021, the largest groups in terms of population size were India (1^st^; 11.3% of all migrants), Poland (2^nd^; 9.1%), Scotland (3^rd^; 8.0%), Pakistan (4^th^; 7.7%), and Romania (5^th^; 6.6%). While India, Scotland, and Pakistan all featured in the top five back in 2001; Poland (19^th^) and Romania (34^th^) have experienced prolific increases in population sizes over the past twenty years (Poland +1173% and Romania +7378% in 2021 versus 2001).

**Online** **S3 File** reorientates the results in Figs 2 and 3 to focus explicitly on the magnitude of the differences in **DFLE** and **LE** among immigrants compared to the England and Wales-born population. The immigrant groups remain in the same order as the original figures. These figures allow easier interpretation of the expected number of years difference between groups. For example, in 2020−22 we can see that Somalia-born women had the highest life expectancy of any group, 4.9 years more than England and Wales-born women, however this came alongside approximately 1 year less **DFLE**. Conversely, we can see higher **DFLE** for women born in France 3.7 years more than the majority population. Lowest measured **DFLE** can be seen amongst Bangladeshi women with 6.6 fewer years expected to be disability-free. Men born in Iran have the highest remaining **LE** in 2020−22, 3.9 additional years compared to England and Wales-born, at the other end men born in Lithuania face a considerably lower **LE** with 3.9 fewer years expected to live after age 50 compared to men born in England and Wales. **DFLE** is especially high for men born in Hong Kong who can expect 5.5 additional years disability-free compared to the majority population. Much like the results for women Bangladeshi men have the lowest **DFLE** experiencing 4.2 fewer years disability-free compared to the England and Wales-born population.

## Discussion

In this study we sought to move beyond single-outcome estimates of immigrant health and contribute novel estimates of disability-free life expectancy and life expectancy at the most granular country-of-birth level across three time points in England & Wales. We have reported substantial heterogeneity in the health of immigrants. Overall, most groups can be classified as experiencing a classic and consistent “Healthy Immigrant Effect” (**HIE**). These groups live longer lives with fewer years spent with disability compared to non-migrants. Simultaneously, we identified several vulnerable groups that were living longer lives with fewer years spent disability-free—a “Migrant Morbidity Mortality Paradox” (**MMP**) [16,30]. Namely, this included men and women born in Turkey and women born in Somalia, Iraq, Afghanistan and Iran. Importantly, we also showed that the health situation of immigrant groups can change over time. Men and women born in Pakistan, Bangladesh and Jamaica, and men born in Somalia all transitioned from living *longer* lives with fewer years spent disability-free to living *shorter* lives with fewer years spent disability-free when compared to non-migrants. Equally, the health situation of immigrant populations can *improve* over time, with men and women born in India and Kenya having transitioned from living shorter lives with fewer years spent disability-free to longer lives with more years spent disability-free in two decades.

For the majority of our groups, we reported a **HIE**. We view this as convincing support for positive selection in migration [46]. Most members of these groups have arrived in the past two decades with population age sex-structures that are young and have shown little signs of ageing between 2001 and 2021, **Online** **S2 File**. This suggests a continual inflow of recently arrived, young adults—two factors associated with a pronounced **HIE** [47]. More than that, these groups are known to be more positively selected, and thus have larger probabilities of being highly-qualified, working in highly-skilled occupations, and being economically active and ultimately better health [46,48]. This is what we anticipate among immigrants born in EU-15 countries, the United States, Canada, Australia, New Zealand, and South Africa compared to non-migrants. The same is also true of immigrants born in non-Western countries that experience a **HIE** too, for example Nigeria, Kenya, Sri Lanka, China, although their socioeconomic advantage is typically limited to being highly-qualified. Beyond selection effects, many of these groups are majority white and experience less racism and discrimination than other groups which may contribute to better health [49].

Migrants born in Northern Ireland and Scotland and male migrants born in the Republic of Ireland experienced a consistent **UIE** in 2000–2, 2010–12 and 2020–22. In addition, women born in the Republic of Ireland show very close to this outcome, yet in 2020–22 are living marginally shorter lives in marginally better health. These groups have older population age structures concentrated at ages 60–84 (**Online** **S2 File**). Their population age structures have also aged considerably between 2001 and 2021, suggesting that few people have arrived from these countries in the past two decades. Older migrants in these groups have been shown to be negatively selected, with moves driven by poverty and ill-health, facilitated by a lack of legal barriers (owing to the *Common Travel Area* for the Republic of Ireland and the fact that moves between Scotland, Northern Ireland, England, and Wales are essentially internal migrations). Additionally, older migrants in these groups typically faced socioeconomic inequality after arriving, and are well-known to have higher rates of smoking and alcohol consumption, worse diet, and a more sedentary lifestyle [50].

The case for men born in Poland and Lithuania, two other groups that experienced a consistent **UIE**, is different. The age structures of these two groups are young (**Online** **S2 File**) and the migration is recent, owing to their countries’ mid-2000s accession to the EU. Yet, unlike other recently arrived groups that experienced a **HIE**, men born in Poland show high levels of socioeconomic disadvantage with relatively poor returns on education and overrepresentation in routine and manual occupations [51]. This differs from women born in Poland (who experience a **HIE**) who are socioeconomically more comparable to non-migrant women and women born in other high-income countries. The results for Poland and Lithuania may also reflect negative selection. The UK was one of only three EU countries *not* to impose transitional restrictions on EU-accession workers, so they could live and work immediately in the UK without work permits or quotas [52]. This *may* have resulted in the arrival of less-selected men to the UK who would not have received work permits elsewhere. Given that men in these two groups are concentrated in low-skilled, manual occupations in manufacturing, agriculture, and logistics [52], their shorter lives in worse health may also reflect a consequence of their physical daily working conditions and/or comparatively unsafe working environments.

Notably, some groups have seen change over time in their health situation. We identify those from India and Kenya as showing a notable *improvement* in their health situation across the three time points; by 2020–22 they exhibit a **HIE.** Our analysis is not equipped to test if this is due to an improvement in the health situation of existing migrants that were already present in 2001 and/or the arrival of healthier migrants between 2001 and 2021. We can infer from the population structures in **Online** **S2 File** that for Kenya the population seems to be ageing in place implying that the health of this group has improved relative to natives over the time points. It should be noted though that the relative disadvantage in **DFLE** compared to the England and Wales-born at earlier time points was small. Conversely, we do observe increases in shares of young adult Indian immigrants (25–49) between 2001 and 2021 (**Online** **S2 File**). This compositional change is consistent with a classic **HIE**, which is typically strongest among young adult immigrants and/or newly arrived immigrants [47]. The patterns found for India *may* reflect a change in selectivity of the migration streams towards highly skilled, highly select immigrants. These immigrants now outnumber older migrants, whose health might have remained poor relative to native populations but is masked at group level by the good health of these newer arrivals.

Some groups that experience a **UIE** in 2021 had previously displayed an **MMP**. This *deterioration* in their health situation is particularly prevalent for immigrants born in Pakistan, Bangladesh and Jamaica. These groups have experienced some compositional change with younger, more recent arrivals but it has not occurred to the same extent as for India. Whilst Pakistan and Bangladesh saw increases in the shares of young adult migrants (aged 25−49), they equally witnessed sizeable increases in shares of older migrants aged 50−74 as well. This could represent the “ageing-in-place” of migrants between 2001 and 2021 and/or the arrival of recent, unhealthy older adult migrants. Jamaica’s population age structure, meanwhile, aged considerably between 2001 and 2021, with visible reductions in shares of young adult migrants (**Online** **S2 File**). Tellingly, these represent the groups in UK society which face large socioeconomic disadvantages in every life domain [48,53]. These disadvantages may accumulate over the life course, along with experiences of discrimination [49,54] resulting in an acceleration of health decline, so-called ‘weathering’ [55,56]. Moreover, structural issues related to healthcare access for these groups may have led to health deterioration as they have aged [57,58]. However, these mechanisms cannot be explicitly tested using this aggregate data. Immigrants born in Pakistan, Bangladesh, and Jamaica were disproportionately impacted by death from COVID-19 in the UK [59]. Given that these groups were all living longer lives with fewer years spent disability-free *before* COVID-19 (i.e., an **MMP**; which may in itself have placed them at additional risk of infection and death from COVID-19), it may be that our results for these groups are a “period” effect (as opposed to a deterioration) and the life expectancy of these groups may recover in the future—if it has not done so already.

Groups that are highly represented in showing the **MMP** in 2021 are men and women born in Turkey and women born in Afghanistan, Iraq, Iran and Somalia. Men born in Afghanistan, Iraq, and Iran show a **HIE** in 2021, for Somalia it is an **UIE**. All have shown evidence of the **MMP** for men at previous time points, and even in 2021 the proportion of life spent without disability is lower than the England and Wales-born. The age structures of these groups are young and has remained so between 2001 and 2021, with most immigrants concentrated between ages 30 and 54 (**Online** **S2 File**). We also know that recent migrant inflows from these countries have been dominated by individuals seeking asylum in the UK. Refugees face distinct health challenges that labour migrants do not, not least in relation to trauma, stress and mental health, communicable diseases, malnutrition, sexual and reproductive health risks, and an inability to fully access healthcare in the new host country [60–62]. These additional risks may help to explain their lower number of years spent disability-free. Previous research from Denmark has reported that refugees have lower mortality risks despite their increased burden of multimorbidity compared to non-migrants [29].

Overall, our findings are consistent with previous findings that show that living longer lives in worse health than non-migrants is largely limited to immigrants born in low- and middle-income countries living in high-income countries—particularly among women born in said countries [16], which suggests overlap with the well-established “gender paradox” in morbidity and mortality [63]. Speculatively, we suggest that the different migration pathways, labour market integration and care burdens experienced by migrant women compared to their male counterparts can materialise in a *double burden* amongst those from the most marginalised migrant backgrounds.

Our research has several strengths. The use of total population data means that we have been able to examine health and health inequalities at lowest level country-of-birth, including for many countries that previous work in the UK has never been able to identify and analyse individually. This adds an unprecedented level of country-of-birth detail to an evidence base that is regularly limited to estimating health expectancies among highly-aggregated categorisations of immigrants due to a lack of available data (e.g., all “foreign-born” and “European” versus “non-European”) [16]. Additionally, we have implemented a repeated cross-sectional temporal element that has allowed us to show whether and how the health situation of specific immigrant groups has changed between 2000−2, 2010−12 and 2020−22. The progression beyond single outcomes (such as mortality as a sole indicator of health) is a strength; we integrate disability and mortality which represents a very important step forward in identifying health inequalities (e.g., living longer lives in worse health) that would not otherwise be identifiable through morbidity or mortality measures alone.

Simultaneously, various limitations to the work must also be acknowledged. **First**, our research design is descriptive. While we say a lot about *how* the **DFLE** and **LE** of a large number of immigrant groups differ from one another and from non-migrants, we cannot say anything about *why* they do, nor do we test any potential mechanisms. Moreover, we cannot determine whether these patterns reflect compositional changes in the immigrant population – through the arrival of newer, healthy/unhealthy immigrants, or the exit of negatively selected migrants (“salmon bias effect”) – and/or the changing health circumstances of existing immigrants through processes such as weathering and acculturation, shifts in socioeconomic circumstances, changes in access to health care, or impacts of public health policies, among other things.

**Second**, although the use of unaggregated country of birth represents an exceptional strength of the study, it is important to acknowledge that heterogeneity persists within country of birth categories according to other migration characteristics (e.g., age at arrival, length of stay, reason for arrival, nationality) and socioeconomic background (e.g., education, income) within subpopulations of the same countries of birth that might lead to different outcomes.

**Third**, we have previously specified in the methods section why we have adopted a prevalence-based approach to **DFLE**. Nevertheless, we acknowledge that prevalence is a static measure that assumes age-specific prevalence of disability is fixed and time-invariant. Thus, we cannot distinguish between changes driven by transitions in incidence and recovery rates, and assume that disability is independent from survival [43].

**Fourth**, although our analyses draw on official population-wide data sources and incorporate ONS imputation and coverage adjustment procedures, they remain subject to potential numerator–denominator bias because mortality counts and population denominators are derived from different sources (death registrations and censuses, respectively). Numerator bias may arise if migrant deaths occurring abroad are not captured in England and Wales mortality statistics. Given migrants’ greater transnational ties and higher rates of circular migration, they may be more likely than the England and Wales-born population to die outside the country. As the ONS has no systematic mechanism for recording deaths of residents abroad, such deaths are likely undercounted, potentially leading to an underestimation of migrant mortality. Denominator bias is also possible because census-based population estimates may both undercount and overcount migrant populations. Undocumented migrants and those spending extended periods abroad at the time of enumeration may be missed by the census, while some individuals may retain residence status to preserve access to services or entitlements, resulting in over-coverage. Since foreign-born populations are disproportionately represented among those missed by the census, these issues may be particularly relevant for migrant groups. However, the overall direction and magnitude of any resulting bias remain uncertain and are not well understood empirically [47].

**Fifth**, we must additionally highlight the fact that the health and death data for 2020−22 encompasses COVID-19 at its peak. We have already discussed the potential influence that COVID-19 had on the mortality risks of migrants born in Pakistan, Bangladesh, Jamaica, and Somalia. Equally, however, COVID-19 may have affected self-reports of disability (through the selective deaths of those with disabilities before the census and through changes in perceptions of disability among those people who survived) and **DFLE,** and may have done so differentially across different immigrant populations [37].

**Sixth**, the question on limiting long-term illness has changed over time from two to three categories (between 2001 and 2011) and become a two-stage question in 2021 [37]. Nevertheless, the measure is still considered to be broadly comparable over time [64,65].

Overall, we report important heterogeneity in the life expectancy and disability-free life expectancy of immigrants, identifying a substantial number of countries of birth that experience a “Healthy Immigrant Effect” in their life and disability-free life expectancy, combined with a smaller set of vulnerable groups that are living longer lives with more disability (a “Migrant Morbidity Mortality Paradox”), shorter lives with more disability (an “Unhealthy Immigrant Effect”), or indeed transitioning from the former to the latter. A large and diverse number of groups exhibit a “Healthy Immigrant Effect”, which may have positive implications for economic growth, labour force participation, public health resilience, and the capacity of public health services. However, the groups exhibiting a “Migrant Morbidity Mortality Paradox” or “Unhealthy Immigrant Effect” represent some of the largest immigrant groups in terms of their population size (Poland [men], Scotland, Pakistan, Lithuania [men], the Republic of Ireland, and Bangladesh). Together, these countries accounted for 38.8% of all immigrants in England & Wales in 2021. These groups will require additional years of care, could place unique pressures on local and national healthcare systems, and may require tailored, targeted public health interventions to help alleviate their higher burden of disability and/or mortality. We should seek to understand why we observe such heterogeneity across country of birth, using data and research designs more explicitly set up to test explanations such as selection effects, socioeconomic inequality, access to health care, and lifestyle [16].

## Supporting information

S1 File Online File S1. Additional tables of life expectancy and disability-free life expectancy and healthy life expectancy at age 0, age 20 and age 50. (XLSX)

S2 File Online File S2. Figures and tables relating to the population age structure and disability prevalence across groups in England and Wales. (DOCX)

S3 File Online File S3. Figures showing the differences in LE and DFLE at age 50 between immigrant groups and the England and Wales-born. (DOCX)
