# Supplementary material for: Immigrant life expectancy and disability-free life expectancy by detailed country of birth in England and Wales: A total population, repeated cross-sectional approach
Source: PLoS One. 2026 Aug 19;21(8):e0356481. doi: 10.1371/journal.pone.0356481 (PMC13489414; doi:10.1371/journal.pone.0356481)
Supplement: Supplementary file 1 [file pone.0356481.s002.docx]

**Online file S2**

 **Fig S2.1.** Population age-sex structures by lowest level country of birth, 2000-2 and 2020-22.

**Source:** Office for National Statistics commissioned tables: **C1471** - Bespoke country of birth by general health by sex by age; **CT21_0404 -** Country of birth by bespoke general health by sex by age

**Notes:** [1] age bands run from 20-24 up to 90+; [2] x-axis standardised at 100,000 people [3] population age-sex structure for England & Wales provided below for comparative purposes; the x-axis for England & Wales is NOT the same as the above panel but is instead set at 2,000,000.

**Table S2.1.** Ranked population size by country of birth in 2001, 2011 and 2021 (including percentage change to previous timepoints).

**Source:** Office for National Statistics commissioned tables: **C1471** - Bespoke country of birth by general health by sex by age; **CT1334** - Bespoke country of birth by general health by sex by age; and **CT21_0404 -** Country of birth by bespoke general health by sex by age.

**Table S2.2.** Number of cases of poor self-rated health, disability, and mortality by country of birth in 2001, 2011, and 2021; women.

**Source:** Office for National Statistics commissioned tables: **C1471** - Bespoke country of birth by general health by sex by age (SRH, 2001); **CT1334** - Bespoke country of birth by general health by sex by age (SRH, 2011); and **CT21_0404 -** Country of birth by bespoke general health by sex by age (SRH, 2021); **C1472** - Bespoke country of birth by limiting long-term illness by sex by age (LLTI, 2001); **CT1335** - Bespoke country of birth by long-term health problem or disability by sex by age (LLTI, 2011); and custom data table “England and Wales, Age (91 categories), Country of birth (60 categories), Sex (2 categories) and Disability (3 categories)” downloaded from “[Create a custom dataset - Office for National Statistics](https://www.ons.gov.uk/datasets/create)”; (LLTI, 2021).

**Table S2.3.** Number of cases of poor self-rated health, disability, and mortality by country of birth in 2001, 2011, and 2021; men

**Source:** Office for National Statistics commissioned tables: **C1471** - Bespoke country of birth by general health by sex by age (SRH, 2001); **CT1334** - Bespoke country of birth by general health by sex by age (SRH, 2011); and **CT21_0404 -** Country of birth by bespoke general health by sex by age (SRH, 2021); **C1472** - Bespoke country of birth by limiting long-term illness by sex by age (LLTI, 2001); **CT1335** - Bespoke country of birth by long-term health problem or disability by sex by age (LLTI, 2011); and custom data table “England and Wales, Age (91 categories), Country of birth (60 categories), Sex (2 categories) and Disability (3 categories)” downloaded from “[Create a custom dataset - Office for National Statistics](https://www.ons.gov.uk/datasets/create)”; (LLTI, 2021).
