# Supplementary material for: Immigrant life expectancy and disability-free life expectancy by detailed country of birth in England and Wales: A total population, repeated cross-sectional approach
Source: PLoS One. 2026 Aug 19;21(8):e0356481. doi: 10.1371/journal.pone.0356481 (PMC13489414; doi:10.1371/journal.pone.0356481)
Supplement: Supplementary file 2 [file pone.0356481.s003.docx]

**Online File S3**

**Fig S3.1.** Differences in Life Expectancy and Disability-Free Life Expectancy at age 50, Compared to England & Wales-Born by Country of Birth, for women

Notes: **(1)** Origin countries are ranked within 2020-22 typologies by life expectancy in 2020-22

Source: Authors’ calculations based upon *Office for National Statistics* information licensed under the *Open Government Licence v.3.0*.

**Fig S3.2:** Differences in Life Expectancy and Disability-Free Life Expectancy at age 50, Compared to England & Wales-Born by Country of Birth, for men

Notes: **(1)** Origin countries are ranked within 2020-22 typologies by life expectancy in 2020-22

Source: Authors’ calculations based upon *Office for National Statistics* information licensed under the *Open Government Licence v.3.0*.
