# Supplementary material for: Immigrant life expectancy and disability-free life expectancy by detailed country of birth in England and Wales: A total population, repeated cross-sectional approach
Source: PLoS One. 2026 Aug 19;21(8):e0356481. doi: 10.1371/journal.pone.0356481 (PMC13489414; doi:10.1371/journal.pone.0356481)
Supplement: Supplementary file 3 [file pone.0356481.s005.docx]

Dear Editors,

Re: Resubmission of manuscript — *Inequalities in life expectancy and disability-free life expectancy by lowest level country of birth in England and Wales: a total population, repeated cross-sectional approach*

We are pleased to resubmit the above manuscript for consideration in PLOS ONE. We are grateful to the reviewers for their thorough and constructive comments, which we believe have strengthened the manuscript considerably.

In response to the reviewers’ feedback, the major changes to the manuscript include:

Firstly, a new title to avoid any confusion regarding spatial inequalities. The new title we propose is “Immigrant life expectancy and disability free life expectancy by detailed country of birth in England and Wales: a total population, repeated cross-sectional approach”.

Additionally, we have modified the abstract. Developed the manuscript to incorporate discussion of additional mechanisms that the reviewers highlighted. Been clearer with the description of how the Sullivan method was implemented and how the categorisation into typologies were done. Lastly, we include two new figures in the Online Files which show the raw difference in LE and DFLE between natives and Each immigrant group. This hopefully means differences can be interpreted more meaningfully.

A tracked-changes version of the manuscript is attached to facilitate review of all modifications. A full point-by-point response to each reviewer comment follows below, with our responses shown in **bold**.

We hope that the revised manuscript is now suitable for publication in PLOS ONE and look forward to hearing from you.

Yours sincerely,

The Authors

Reviewers' comments:

Reviewer's Responses to Questions

**Comments to the Author**

1. Is the manuscript technically sound, and do the data support the conclusions?

The manuscript must describe a technically sound piece of scientific research with data that supports the conclusions. Experiments must have been conducted rigorously, with appropriate controls, replication, and sample sizes. The conclusions must be drawn appropriately based on the data presented.

Reviewer #1: Partly

Reviewer #2: Yes

2. Has the statistical analysis been performed appropriately and rigorously?

Reviewer #1: Yes

Reviewer #2: Yes

3. Have the authors made all data underlying the findings in their manuscript fully available?

The [PLOS Data policy](https://eur01.safelinks.protection.outlook.com/?url=http%3A%2F%2Ftrack.editorialmanager.com%2FCL0%2Fhttp%3A%252F%252Fwww.plosone.org%252Fstatic%252Fpolicies.action%2523sharing%2F1%2F010f019e2a1047f0-a301de67-ef8b-4dbc-a6e5-b37aa1a421fd-000000%2F2rSNDAexAfoCLbPY-Q_N0-bQDzQGfekTR4jGK8nm8NU%3D258&data=05%7C02%7Cj.i.harrison%40salford.ac.uk%7C03c5e85ca29e4317ea6208deb241147f%7C65b52940f4b641bd833d3033ecbcf6e1%7C0%7C0%7C639144190070555724%7CUnknown%7CTWFpbGZsb3d8eyJFbXB0eU1hcGkiOnRydWUsIlYiOiIwLjAuMDAwMCIsIlAiOiJXaW4zMiIsIkFOIjoiTWFpbCIsIldUIjoyfQ%3D%3D%7C0%7C%7C%7C&sdata=8Malvo%2FW2oWZ9ROhyqPVZJV1YPidVjCTWtHdDw9%2B3yA%3D&reserved=0) requires authors to make all data underlying the findings described in their manuscript fully available without restriction, with rare exception (please refer to the Data Availability Statement in the manuscript PDF file). The data should be provided as part of the manuscript or its supporting information, or deposited to a public repository. For example, in addition to summary statistics, the data points behind means, medians and variance measures should be available. If there are restrictions on publicly sharing data—e.g. participant privacy or use of data from a third party—those must be specified.

Reviewer #1: Yes

Reviewer #2: Yes

4. Is the manuscript presented in an intelligible fashion and written in standard English?

PLOS ONE does not copyedit accepted manuscripts, so the language in submitted articles must be clear, correct, and unambiguous. Any typographical or grammatical errors should be corrected at revision, so please note any specific errors here.

Reviewer #1: Yes

Reviewer #2: Yes

5. Review Comments to the Author

Please use the space provided to explain your answers to the questions above. You may also include additional comments for the author, including concerns about dual publication, research ethics, or publication ethics. (Please upload your review as an attachment if it exceeds 20,000 characters)

Reviewer #1: Thank you very much for the opportunity to review the manuscript entitled “Inequalities in life expectancy and disability-free life expectancy by lowest level country of birth in England and Wales: A total population, repeated cross-sectional approach.”

This is an ambitious and policy-relevant study drawing on exceptionally rich census and mortality data. The disaggregation to lowest-level country of birth is a major empirical contribution, and the typology in Table 2 (HIE, UIE, MMP, SLBH) is helpful for interpretation. Overall, the paper makes an important contribution to UK and international research on migrant health inequalities. Below, I outline the main points for consideration.

Major points
• The study uses country of birth as the primary analytic axis. While this is understandable given the data constraints, it risks essentialising national-origin groups. Greater acknowledgment of within-country-of-origin heterogeneity (e.g., by migration reason, SES, ethnicity, years since migration) would strengthen the conceptual framing.

**Thank you for this comment. While the use of unaggregated country of birth represents an exceptional strength of the study, we agree that heterogeneity exists within country of birth categories that needs to be highlighted in the paper in order to avoid essentialising national-origin groups. In the limitations section (page 21, line 449), we have added the following text:**

*“Second, although the use of unaggregated country of birth represents an exceptional strength of the study, it is important to acknowledge that heterogeneity persists within country of birth categories according to other migration characteristics (e.g., age-at-arrival, length of stay, reason for arrival, nationality) and socioeconomic background (e.g., education, income) within subpopulations of the same countries of birth that might lead to different outcomes.”*

**We hope the inclusion of this text in the limitations section is sufficient.**

• The discussion could engage more explicitly with structural frameworks beyond MMP, including the weathering hypothesis, differential healthcare access, and racism/discrimination. This is particularly relevant to the interpretation of COVID-19, which may have amplified existing structural inequalities rather than functioning solely as a temporary period effect.

**Included the following in the discussion Page 19 line 389**

*“Tellingly, these represent the groups in UK society which face large socioeconomic disadvantages in every life domain (48,53), these disadvantages may accumulate over the life course, along with experiences of discrimination (49,54) resulting in an acceleration of health decline, so called ‘weathering’ (55,56). Moreover, structural issues related to healthcare access for these groups may have led to health deterioration as they have aged (57,58). However, these mechanisms cannot be explicitly tested using this aggregate data.”*

• The HIE is interpreted largely through positive selection. While important, selection should not be treated as the only explanation. It would help to distinguish more clearly between health selection at arrival and post-migration health trajectories, and to consider structural constraints, such as migration regimes, legal precarity, and racism/discrimination.

**We agree with you that these mechanisms may also be operating. While we firmly believe these explanations should be distinguished between. Since we do not use individual level data, we do not have information about health status on arrival or migration regimes that people arrive under, thus we are limited in exploring this. We see selection as the obvious explanation for many of these groups since the age structure does not change over time for many of these groups. Implying that it is different young immigrants who are positively selected on health who are present at each time point. We have added the below to our discussion of the HIE groups to highlight that many of them are majority white and thus do not experience racism which as a potential explanator.**

**“**Beyond selection effects, many of these groups are majority white and experience less racism and discrimination than other groups which may contribute to better health (49).”

• The limitations section should more clearly address compositional issues (e.g., salmon bias, years since migration, mortality selection) and the static assumption of prevalence-based health expectancy. Given the claims about change over time, readers may otherwise over-interpret compositional shifts as causal change.

**Thank you for this comment. In the main figures, we have removed the “improving” and “deteriorating” groups to help avoid misinterpretation as causal change. In the limitations section, we have added the following about the issue of composition specifically (Page 21, line 439):**

***“****Simultaneously, various limitations to the work must also be acknowledged.* ***First****, our research design is descriptive. While we say a lot about how the* ***DFLE*** *and* ***LE*** *of a large number of immigrant groups differ from one another and from non-migrants, we cannot say anything about why they do, nor do we test any potential mechanisms. Moreover, we cannot determine whether these patterns reflect compositional changes in the immigrant population - through the arrival of newer, healthy/unhealthy immigrants, or the exit of negatively selected migrants (“salmon bias effect”)- and/or the changing health circumstances of existing immigrants through processes such as weathering and acculturation, shifts in socioeconomic circumstances, changes in access to health care, or impacts of public health policies, among other things.”*

**Regarding the static assumption of prevalence, we have added the following (Page 22, line 455):**

***“Third****, we have previously specified in the methods section why we have adopted a prevalence-based approach to* ***DFLE****. Nevertheless, we acknowledge that prevalence is a static measure that assumes age-specific prevalence of disability is fixed and time-invariant, cannot distinguish between changes driven by transitions in incidence and recovery rates, and assumes that disability is treated as independent from survival (43).”*

**We hope that this expansion of the limitations section is sufficient and more accurately reflects the limitations associated with the analysis in the main paper.**

• The HIE/UIE/MMP/SLBH typology is useful but may oversimplify when differences are marginal. Please clarify the decision rule for classification and explicitly indicate whether the confidence interval overlaps with the reference group.

**We agree that it is important to clarify the decision rule for classification and thank the reviewer for identifying this oversight. The other reviewer also identified this issue. We agree with both reviewers that the classification is useful but concede that more transparency is necessary. Through discussing this comment, we have opted to use and outline a somewhat simpler approach. Importantly, we welcome further input from both reviewers if they think an alternate classification approach is necessary. Originally, we had made this classification based upon the point estimates (given the use of comprehensive total population data we believe that the estimates alone are substantive), alongside a threshold approach for the different years. Specifically, if a Healthy Immigrant Effect was reported in >=2 of 3 census years, we classified those groups as “HIE”. Nonetheless, while conceiving this classification approach, we had extensive conversations about the role of (a) the size of differences in DFLE and LE to the England & Wales-born, (b) the role of confidence intervals, and (c) the ratio of total life spent without disability. Despite settling upon this approach, we remained uncomfortable with threshold cases where 2001 and 2011 were consistent but 2021 (i.e., the recent estimate) was not.**

**In this revision, we have decided to simplify the classification about by removing the temporal element from the classification and basing it upon point estimates for 2021 alone. This helps to remove complications of the threshold approach but also places an emphasis on the most recent situation of migrants, which we believe is more relevant and timelier policy-wise. Additionally, we have introduced two additional figures as an online file that calculate and show differences in DFLE and LE among migrant compared to the England & Wales-born that more intuitively show the magnitude of the difference in DFLE and LE to the England & Wales-born and whether or not confidence intervals overlap with the reference.**

**We have introduced the following text in the manuscript (Page 8, line 143):**

*“After deriving the estimates for* ***LE50*** *and* ***DFLE50*** *for each country of birth at each time point, we compare them to the estimate of those born in England and Wales. Based on these comparisons, migrant groups were categorised according to whether LE and DFLE were higher or lower than the native-born population in 2020-22. As shown in Table 2 this results in four exclusive categories. For example, groups with both higher LE and higher DFLE than the England and Wales-born population were classified as exhibiting a Healthy Immigrant Effect (HIE).*

*We choose the 2020-22 period because it represents the most recent and policy-relevant period, providing the clearest picture of the contemporary health situation of migrant populations. However, it is important to acknowledge that some groups have changed over time.*

*Our categorisation is based solely on differences in point estimates. We adopted this approach for simplicity and because the analyses are based on comprehensive population data, making the observed values substantively meaningful. We recognise, however, that alternative approaches could have been used, including consideration of the magnitude of differences, overlap in confidence intervals, or the proportion of remaining life spent disability-free. Consequently, the classification involves an element of subjectivity. Nevertheless, we believe that this framework is both useful and necessary for summarising patterns across the large number of countries included in the analysis. Confidence intervals are presented in the figures and in Online File S1 to allow readers to assess the uncertainty surrounding the estimates.”*

**We hope this is sufficient. If not, we warmly welcome suggestions from reviewers.**

• Gender differences warrant deeper theorisation, particularly regarding labour market integration, care burdens, and migration pathways.

**Included the following in the discussion (Page 20 line 417)**

*“Overall, our findings are consistent with previous findings that show that living longer lives in worse health than non-migrants is largely limited to immigrants born in low and middle-income countries living in high-income countries—particularly among women born in said countries (16), which suggests overlap with the well-established “gender paradox” in morbidity and mortality (63). Speculatively we suggest that the different migration pathways, labour market integration and care burdens experienced by migrant women compared to their male counterparts can materialise in a double burden amongst those from the most marginalised migrant backgrounds.”*

Minor points

• Clarify the rationale for selecting age 50.

**Thank you for this comment. We selected age 50 because this is a common age at which healthy/disability-free life expectancies are calculated in previous studies. We note, however, that we also include estimates from birth and age 20 in Online File S1. We refer readers to these additional estimates on page 8 line 139:**

***“****To maximise our comparability with previous research, we report results for life and disability-free life expectancies at age 50 (****LE50*** *and* ***DFLE50****). Estimates for expectancies from birth and age 20 are in* ***Online File S1****.****”***

**We also briefly describe these estimates and how they differ (or not) to the England & Wales-born on page 13 line 254 here:**

*“From* ***online file S1****, we report that healthy life expectancy (****HLE****) is typically 4-6 years higher among men and 6-8 years higher among women. The patterns are consistent with* ***DFLE*** *across countries of birth. All* ***HIE*** *groups that have higher* ***LE*** *and* ***DFLE*** *also exhibit a higher* ***HLE*** *than non-migrants. Similarly,* ***MMP*** *groups that have higher* ***LE*** *and lower* ***DFLE*** *also have lower* ***HLE*** *and/or lower % of years spent in good health than non-migrants do. This is unsurprising given how much self-rated health and limiting long-term illness are correlated (44). Simultaneously,* ***SRH*** *and* ***LLTI*** *do capture different aspects of health.* ***LLTI*** *provides a more objective, function-focused measure of (chronic) morbidity and daily limitations, while SRH provides a more subjective and holistic view of wider health (including issues that are yet to be diagnosed), its perceptions, and determinants (45).* ***Online file S1*** *reinforces our findings, adds valuable additional evidence based upon a complementary measure of health, and shows that nearly all instances of* ***HIE****,* ***UIE*** *and* ***MMP*** *can be extended to broader definitions of health. Additionally, aside from increases in the values of* ***LE*** *and* ***DFLE*** *themselves, for* ***LE0****,* ***LE20****,* ***DFLE0*** *and* ***DFLE20****, group membership remains consistent by birth country.”*

• Explain how small cell suppression and minimum death counts were handled.

**For the self-rated health and limited long-term illness (i.e., census) data, the ONS uses targeted recording swapping on the census microdata combined with cell key perturbation at the table-generation stage. These record swaps are exclusively limited to changes between different geographic areas and or housing types (i.e., communal establishments). Given that we only analyse the data at the national level this should not have an impact upon our results. At the table stage, for the smallest counts (<5) the ONS performs cell perturbation, adding a small amount of “noise” to the cell in question to prevent individuals requesting overlapping tables that can be used to recover unique cases. Therefore, no cells are supressed. This might have a minor impact upon our results—albeit detailed checks of the data reveal very few cell counts less than 5 even in the smallest migrant populations.**

**For minimum death counts, the approach is slightly different. ONS implements a lighter touch approach when ad-hoc/bespoke tables are requested that require the analyst compiling the tables to negotiate with the data requester to avoid having to supress small numbers. In our case, this including use of 5-year age groups rather than single year age groups), larger geographic areas (i.e., all of England & Wales) and the aggregation of smallest groups where cell counts <3 were recorded (these aggregated groups e.g., Northern Africa, Central America) were provided to us but not included in the analysis—owing to a desire to focus on detailed birth country.**

• Proofread carefully (e.g., “leading to suggests of a disconnect” in the abstract).

**The whole article has been proofread and this error in the abstract has been corrected.**

Overall, this is a strong and valuable contribution. Addressing the points above would further strengthen its theoretical clarity and methodological transparency.

Reviewer #2: I would like to thank the authors for this interesting manuscript. The paper addresses an important topic and has the potential to make a useful contribution to the literature on migrant health, life expectancy, and disability-free life expectancy. The use of detailed country-of-birth data across three time points is a clear strength. However, I believe that several major issues need to be addressed before the manuscript can be considered for publication.

1. Title and focus of the paper
The full title suggests that the paper focuses primarily on spatial inequalities in life expectancy and disability-free life expectancy in England and Wales. However, the short title and, more importantly, the manuscript itself focus mainly on inequalities by country of birth / immigrant background. I would therefore recommend revising the title so that it more accurately reflects the actual content and emphasis of the paper.

**The original title of the paper is:**

*“Inequalities in life expectancy and disability free life expectancy by lowest level birth country in England & Wales: a total population, repeated cross-sectional approach”.*

**It was not our intention with this title to suggest that were focusing on spatial inequalities—however, we understand why the title might be interpreted in that way given the use of “lowest-level country of birth” which may imply lowest level geography, rather than unaggregated country of birth and does not specify that this is the country of birth of immigrants. With this in mind, we have removed the term “lowest level” and replaced it with “immigrant”. We have also removed the word inequalities, a term which can be associated with spatial inequalities.**

**Now, the new title is :**

*“Immigrant life expectancy and disability free life expectancy by detailed country of birth in England and Wales: a total population, repeated cross-sectional approach”.*

**We hope this change is clearer.**

2. Abstract
The abstract would benefit from substantial revision in terms of flow, clarity, and readability. At present, several terms are not sufficiently clear. For example, it is unclear what is meant by “total population mortality” and “lowest-level country of origin”. It would also be helpful to state how many country-of-birth groups are included in the analysis.
The description of the data should also be clarified. The abstract states that the study uses “total population mortality and census data from 2000–02, 2010–12 and 2020–22”. However, census data refer to a single point in time, whereas mortality data appear to be aggregated over three-year periods around each census. This distinction should be made explicit.
I would also suggest removing the statement “providing the most granular analysis of life expectancy (LE) and disability-free life expectancy (DFLE) in the global evidence base to date” from the middle of the abstract. Instead, the abstract should more clearly state that DFLE is estimated using self-reported limiting long-term illness.
In addition, the term “inequalities” should be defined more precisely. If this refers to absolute or relative differences in LE and DFLE between each immigrant group and the England and Wales-born population, this should be stated explicitly. Finally, the repeated cross-sectional / longitudinal aspect of the study is an important strength, but it is not sufficiently highlighted in the abstract.

**Thank you for these suggestions. The entire abstract has been re-written to consider the above suggestions.**

3. Data sources, missing values, and numerator–denominator bias
The manuscript should explain more clearly how missing values were handled, particularly for country of birth in death registrations and census data, and for health status in the census.

I would suggest discussing whether census undercounting, especially among some migrant groups, may affect the results. Since deaths and population exposures by country of birth come from different sources, there is a potential risk of numerator–denominator bias. This issue deserves more explicit discussion.

**The data we are using has been provided by the Office for National Statistics (ONS) – the national statistical agency of England & Wales whose data is used as the basis for all national demographic estimates.**

**We ordered ad-hoc, harmonized tables from the ONS for self-rated health, limiting long-term illness, and all-cause mortality that were broken down by country of birth, age, and sex. We as the authors have not made any decisions around missing values, except to remove the “missing” country group (a category we derived from the data provided to us that included categories such as “born at sea”, “unknown”, and “inadequately described”). This group accounts for less than 0.1% of the data.**

**ONS produces all technical documentation related to their census and mortality data and this is freely available online. The ONS uses a layered set of methods to deal with missing data, tackling two major issues: item non-response (incomplete answers within a returned form) and unit non-response / under-enumeration (people who never returned a form or were missed entirely). To address these issues, ONS implements item editing (i.e., fixing errors and inconsistencies e.g., a person under age 10 with a university degree). The edit rules are defined in consultation with census users and experts to prevent impossible or highly unlikely combinations of values. ONS also implements item imputation via deterministic editing, nearest neighbour donor imputation and through manual imputation.**

**Regarding numerator-denominator bias, we first discuss the numerator. ONS uses a coverage assessment and adjustment methodology via the Census Coverage Survey (CCS) that enables census population counts to be adjusted for under-enumeration at the national, local, and small area level.** **The CCS is then matched against census records using dual system estimation. Dual system estimation works for sampled areas, and the next step is to use the under-coverage-adjusted totals at sample level to produce whole-population estimates via a ratio estimator. Once the extent of under-coverage is estimated, a statistical adjustment is made. Among migrants specifically, little empirical evidence exists. Guillot et al. (2018) theorised explicitly around this issue in their paper suggesting that undercounting among migrants likely depends upon the mobility of migrants, how much time is spent abroad per year, and when/if they are away (i.e., during collection of census data)—which would lead to under-coverage. Certain vulnerable groups, such as undocumented migrants (although they can conceivably be included at censuses because their inclusion is not based upon registration like in other data sources), are also likely to be undercounted. On the other hand, there may also be incentive to maintain “residence” status in a host country (and be counted in those data sources; e.g., pensions, healthcare)—which would lead to over-coverage. Thus, while there may be undercounting, but there may also be overcounting too.**

**When it comes to mortality registrations, the ONS cite registration delays as the primary source of undercounting. It attempts to address this issue through parallel approaches that involve production of registration-based figures (that are more prone to undercounting) and occurrence-based figures (which allow a significant period of time for registration and are what we use in this paper). There is no reason to believe that there are systematic differences in the delays of the registration of deaths in England & Wales among migrants compared to non-migrants. Of more concern is potential deaths missed through the occurrence of deaths abroad—this is also discussed by Guillot et al. (2018). National vital registration systems are poorly equipped to gather data on residents dying abroad. Transnationality and circular migration implies that migrants are more likely to spend more time abroad per year than non-migrants are, increasing the likelihood of death occurring abroad that get missed from the numerator of migrant mortality rates. As above, however, there is little empirical evidence of this. We do know however that ONS does not systematically capture the deaths of residents abroad, it simply acknowledges the gap as part of its methodology. The registry office does hold whatever voluntary overseas registrations of deaths are submitted via the consulates, but there is no systematic mechanism to count and/or include those who die in another country.**

**All of the above methodology is frequently reviewed and tested. While we have not made these decisions, we agree with the reviewer that it is important to outline how missing data and under-coverage were addressed by ONS before we analysed the data. We have added the following text to the data section (Page 5, line 95):**

*“Prior to the receipt of the data, the Office for National Statistics addresses missing and inconsistent data through fixing inconsistent answers (i.e., “item editing”) and item non-response through imputation (specifically deterministic editing, nearest neighbour donor imputation and manual imputation). It also addresses under-coverage through a combination of a coverage survey (the Census Coverage Survey [CCS], dual system estimation and a final statistical coverage adjustment). These corrections have been developed and validated over successive censuses since 2001. More information about these methodologies are available online (34–36).”*

**We also agree that it is important to identify some of these issues as limitations that the readers need to be aware of when interpreting the findings of our paper. Thus we added the following (page 22, line 460)**

*“Fourth, although our analyses draw on official population-wide data sources and incorporate ONS imputation and coverage adjustment procedures, they remain subject to potential numerator–denominator bias because mortality counts and population denominators are derived from different sources (death registrations and censuses, respectively). Numerator bias may arise if migrant deaths occurring abroad are not captured in England and Wales mortality statistics. Given migrants' greater transnational ties and higher rates of circular migration, they may be more likely than the England and Wales-born population to die outside the country. As the ONS has no systematic mechanism for recording deaths of residents abroad, such deaths are likely undercounted, potentially leading to an underestimation of migrant mortality. Denominator bias is also possible because census-based population estimates may both undercount and overcount migrant populations. Undocumented migrants and those spending extended periods abroad at the time of enumeration may be missed by the census, while some individuals may retain residence status to preserve access to services or entitlements, resulting in over coverage. Since foreign-born populations are disproportionately represented among those missed by the census, these issues may be particularly relevant for migrant groups. However, the overall direction and magnitude of any resulting bias remain uncertain and are not well understood empirically (47).”*

**We hope this explanation, and the inclusion of new text is sufficient.**

4. Small numbers and smoothing at older ages when constructing LT
Have you considered applying any smoothing or correction methods, particularly at older ages? Or was instability at older ages not a substantial issue in these datasets?

**Thank you for this comment. As a sensitivity analysis when we generated the main results, we compared the DFLEs with both the raw and smooth data and differences were negligible (often generating identical DFLEs and LEs). With this in mind, we opted to present the findings with the data “as is”. The lack of impact is largely due to the sheer population size of the data that we have available to us.**

5. Description of the Sullivan method.
The statement “From the values for remaining years of life expectancy we use the Sullivan method to calculate the expected number of years being disability-free given the prevalence of disability” is not sufficiently clear. Please provide a more precise description of how the Sullivan method was implemented. In addition, did you estimate 95% confidence intervals for DFLE? If so, this should be stated explicitly in the Methods section, together with the relevant references.

**We include a description of the Sullivan method in the methods section as follows (Page7, line 130)**

*“We estimated DFLE using the Sullivan method, which is widely used for estimation of healthy or disability free life expectancies (24,28,42). For each age interval the person-years lived were multiplied by the proportion of the population reporting no disability in that age group. These disability-free person-years were summed across remaining ages and divided by the number surviving to estimate DFLE at each age. For the confidence intervals we performed a proportion test around the proportion non-disabled to derive upper and lower estimates of disability prevalence.”*

6. The size of inequalities
Much of the analysis is based on comparisons between immigrant groups and the England and Wales-born population. I would therefore recommend presenting the numeric estimates of the gaps in LE and DFLE, together with corresponding confidence intervals. This would make the comparisons more transparent and help readers assess whether observed differences are meaningful.

**Thank you for this suggestion. We have presented two new figures in Online File S3 that present the magnitude of the gap in LE and DFLE with corresponding confidence intervals. The design and country order of the panel mirrors the two previous figures (Figure 2 and Figure 3) that present the LE and DFLE of different groups. We hope the comparisons are more transparent and intuitive to readers with this additional interpretation. We opt not to include it in the main manuscript as the information presented is the same as figures 2 & 3, but we refer to it directly and present some of the inequalities on Page 14 line 282.**

***“Online file S3*** *reorientates the results in* ***Figs 2*** *and* ***3*** *to focus explicitly on the magnitude of the differences in DFLE and LE among immigrants compared to the England and Wales born population. The immigrant groups remain in the same order as the original figures. These figures allow easier interpretation of the expected number of years difference between groups. For example, in 2020-22 we can see that Somalia born women had the highest life expectancy of any group 4.9 years more than England and Wales born women, however this came alongside approximately 1 year less* ***DFLE****. Conversely, we can see higher DFLE for women born in France 3.7 years more than the majority population. Lowest measured DFLE can be seen amongst Bangladeshi women with 6.6 fewer years expected to be disability free. For men born in Iran have the highest remaining LE, 3.9 additional years compared to England and Wales born, at the other end men born in Lithuania face a considerably lower LE with 3.9 fewer years expected to live after age 50 than men born in England and Wales at 2020-22. DFLE is especially high for men born in Hong Kong who can expect 5.5 additional years disability-free compared to the majority population. Much like the results for women Bangladeshi men have the lowest DFLE experiencing 4.2 fewer years disability-free compared to the England and Wales born population.”*

7. Classification of groups
The classification of groups into HIE, MMP, UIE, and SLBH is useful, but the criteria should be made clearer. In particular, the manuscript states that when differences are marginal or not statistically significant, groups are classified into the “most appropriate category”. This is potentially subjective. I would recommend the authors to explain exactly how these decisions were made and whether classifications are based on statistical significance, point estimates or substantive thresholds.

**Thank you for this comment, which the other reviewer also made. We have responded in detail to this comment earlier in the document.**

8. Supplementary analyses
The supplementary analyses are extensive, but their role in the manuscript is not always clear. Some appear to be sensitivity analyses, while others provide descriptive contextual information. I would recommend distinguishing more clearly between supplementary analyses and sensitivity analyses and retaining in the main text only those results that directly support the conclusions.

**In the methods section we have now subtitled supplementary analyses and sensitivity analyses, separately. Additionally, the analysis of the micro census data which was originally used to contextualise findings has been removed.**
