# Supplementary material for: Immigrant life expectancy and disability-free life expectancy by detailed country of birth in England and Wales: A total population, repeated cross-sectional approach
Source: PLoS One. 2026 Aug 19;21(8):e0356481. doi: 10.1371/journal.pone.0356481 (PMC13489414; doi:10.1371/journal.pone.0356481)
Supplement: Peer review history

# Peer review: Decision Letter 0

**DOI:** 10.1371/journal.pone.0356481.r001

**Authors:** Eyal Bar-Haim

15 May 2026

PONE-D-25-65095

Inequalities in life expectancy and disability free life expectancy by lowest level country of birth in England and Wales: a total population, repeated cross-sectional approach

PLOS One

Dear Dr. Harrison,

Thank you for submitting your manuscript to PLOS ONE. After careful consideration, we feel that it has merit but does not fully meet PLOS ONE’s publication criteria as it currently stands. Therefore, we invite you to submit a revised version of the manuscript that addresses the points raised during the review process.

Both reviewers requested methodological clarifications and a more robust treatment of theoretical issues. Although the required revisions are extensive, I believe the manuscript can be updated in a timely fashion.

Please submit your revised manuscript by Jun 28 2026 11:59PM. If you will need more time than this to complete your revisions, please reply to this message or contact the journal office at plosone@plos.org. When you're ready to submit your revision, log on to https://www.editorialmanager.com/pone/ and select the 'Submissions Needing Revision' folder to locate your manuscript file.

Please include the following items when submitting your revised manuscript:

- A letter that responds to each point raised by the academic editor and reviewer(s). You should upload this letter as a separate file labeled 'Response to Reviewers'.
- A marked-up copy of your manuscript that highlights changes made to the original version. You should upload this as a separate file labeled 'Revised Manuscript with Track Changes'.
- An unmarked version of your revised paper without tracked changes. You should upload this as a separate file labeled 'Manuscript'.

If you would like to make changes to your financial disclosure, please include your updated statement in your cover letter. Guidelines for resubmitting your figure files are available below the reviewer comments at the end of this letter.

If applicable, we recommend that you deposit your laboratory protocols in protocols.io to enhance the reproducibility of your results. Protocols.io assigns your protocol its own identifier (DOI) so that it can be cited independently in the future. For instructions see: https://journals.plos.org/plosone/s/submission-guidelines#loc-laboratory-protocols. Additionally, PLOS ONE offers an option for publishing peer-reviewed Lab Protocol articles, which describe protocols hosted on protocols.io. Read more information on sharing protocols at https://plos.org/protocols?utm_medium=editorial-email&utm_source=authorletters&utm_campaign=protocols.

As the corresponding author, your ORCID iD is verified in the submission system and will appear in the published article. PLOS supports the use of ORCID, and we encourage all coauthors to register for an ORCID iD and use it as well. Please encourage your coauthors to verify their ORCID iD within the submission system before final acceptance, as unverified ORCID iDs will not appear in the published article. Only the individual author can complete the verification step; PLOS staff cannot verify ORCID iDs on behalf of authors.

We look forward to receiving your revised manuscript.

Kind regards,

Eyal Bar-Haim

Academic Editor

PLOS One

Journal Requirements:

When submitting your revision, we need you to address these additional requirements.

1. Please ensure that your manuscript meets PLOS ONE's style requirements, including those for file naming. The PLOS ONE style templates can be found at

https://journals.plos.org/plosone/s/file?id=wjVg/PLOSOne_formatting_sample_main_body.pdf and

https://journals.plos.org/plosone/s/file?id=ba62/PLOSOne_formatting_sample_title_authors_affiliations.pdf

2. Thank you for stating the following financial disclosure:

"This work was funded by the UKRI HEu Guarantee: ERC Starting Grants 2023: APP44827 ‘Living longer in poorer health? Understanding the Immigrant Morbidity Mortality Paradox’ (PARA-MOR)"

Please state what role the funders took in the study.  If the funders had no role, please state: "The funders had no role in study design, data collection and analysis, decision to publish, or preparation of the manuscript."

If this statement is not correct you must amend it as needed.

Please include this amended Role of Funder statement in your cover letter; we will change the online submission form on your behalf.

3. We note that you have indicated that there are restrictions to data sharing for this study. PLOS only allows data to be available upon request if there are legal or ethical restrictions on sharing data publicly. For more information on unacceptable data access restrictions, please see http://journals.plos.org/plosone/s/data-availability#loc-unacceptable-data-access-restrictions.

Before we proceed with your manuscript, please address the following prompts:

a) If there are ethical or legal restrictions on sharing a de-identified data set, please explain them in detail (e.g., data contain potentially identifying or sensitive patient information, data are owned by a third-party organization, etc.) and who has imposed them (e.g., a Research Ethics Committee or Institutional Review Board, etc.). Please also provide contact information for a data access committee, ethics committee, or other institutional body to which data requests may be sent.

b) If there are no restrictions, please upload the minimal anonymized data set necessary to replicate your study findings to a stable, public repository and provide us with the relevant URLs, DOIs, or accession numbers. For a list of recommended repositories, please see

https://journals.plos.org/plosone/s/recommended-repositories. You also have the option of uploading the data as Supporting Information files, but we would recommend depositing data directly to a data repository if possible.

We will update your Data Availability statement on your behalf to reflect the information you provide.

4. We note that there is identifying data in the Supporting Information file Online_file_S1.xlsx. Due to the inclusion of these potentially identifying data, we have removed this file from your file inventory. Prior to sharing human research participant data, authors should consult with an ethics committee to ensure data are shared in accordance with participant consent and all applicable local laws.

Data sharing should never compromise participant privacy. It is therefore not appropriate to publicly share personally identifiable data on human research participants. The following are examples of data that should not be shared:

-Name, initials, physical address

-Ages more specific than whole numbers

-Internet protocol (IP) address

-Specific dates (birth dates, death dates, examination dates, etc.)

-Contact information such as phone number or email address

-Location data

-ID numbers that seem specific (long numbers, include initials, titled “Hospital ID”) rather than random (small numbers in numerical order)

Data that are not directly identifying may also be inappropriate to share, as in combination they can become identifying. For example, data collected from a small group of participants, vulnerable populations, or private groups should not be shared if they involve indirect identifiers (such as sex, ethnicity, location, etc.) that may risk the identification of study participants.

Additional guidance on preparing raw data for publication can be found in our Data Policy (https://journals.plos.org/plosone/s/data-availability#loc-human-research-participant-data-and-other-sensitive-data) and in the following article: http://www.bmj.com/content/340/bmj.c181.long.

Please remove or anonymize all personal information location data ensure that the data shared are in accordance with participant consent, and re-upload a fully anonymized data set. Please note that spreadsheet columns with personal information must be removed and not hidden as all hidden columns will appear in the published file.

5. If the reviewer comments include a recommendation to cite specific previously published works, please review and evaluate these publications to determine whether they are relevant and should be cited. There is no requirement to cite these works unless the editor has indicated otherwise.

[Note: HTML markup is below. Please do not edit.]

Reviewers' comments:

Reviewer's Responses to Questions

**Comments to the Author**

1. Is the manuscript technically sound, and do the data support the conclusions?

Reviewer #1: Partly

Reviewer #2: Yes

**********

2. Has the statistical analysis been performed appropriately and rigorously?

Reviewer #1: Yes

Reviewer #2: Yes

**********

3. Have the authors made all data underlying the findings in their manuscript fully available?

The PLOS Data policy

Reviewer #1: Yes

Reviewer #2: Yes

**********

4. Is the manuscript presented in an intelligible fashion and written in standard English?

Reviewer #1: Yes

Reviewer #2: Yes

**********

Reviewer #1: Thank you very much for the opportunity to review the manuscript entitled “Inequalities in life expectancy and disability-free life expectancy by lowest level country of birth in England and Wales: A total population, repeated cross-sectional approach.”

This is an ambitious and policy-relevant study drawing on exceptionally rich census and mortality data. The disaggregation to lowest-level country of birth is a major empirical contribution, and the typology in Table 2 (HIE, UIE, MMP, SLBH) is helpful for interpretation. Overall, the paper makes an important contribution to UK and international research on migrant health inequalities. Below, I outline the main points for consideration.

Major points

• The study uses country of birth as the primary analytic axis. While this is understandable given the data constraints, it risks essentialising national-origin groups. Greater acknowledgment of within-country-of-origin heterogeneity (e.g., by migration reason, SES, ethnicity, years since migration) would strengthen the conceptual framing.

• The discussion could engage more explicitly with structural frameworks beyond MMP, including the weathering hypothesis, differential healthcare access, and racism/discrimination. This is particularly relevant to the interpretation of COVID-19, which may have amplified existing structural inequalities rather than functioning solely as a temporary period effect.

• The HIE is interpreted largely through positive selection. While important, selection should not be treated as the only explanation. It would help to distinguish more clearly between health selection at arrival and post-migration health trajectories, and to consider structural constraints, such as migration regimes, legal precarity, and racism/discrimination.

• The limitations section should more clearly address compositional issues (e.g., salmon bias, years since migration, mortality selection) and the static assumption of prevalence-based health expectancy. Given the claims about change over time, readers may otherwise over-interpret compositional shifts as causal change.

• The HIE/UIE/MMP/SLBH typology is useful but may oversimplify when differences are marginal. Please clarify the decision rule for classification and explicitly indicate whether the confidence interval overlaps with the reference group.

• Gender differences warrant deeper theorisation, particularly regarding labour market integration, care burdens, and migration pathways.

Minor points

• Clarify the rationale for selecting age 50.

• Explain how small cell suppression and minimum death counts were handled.

• Proofread carefully (e.g., “leading to suggests of a disconnect” in the abstract).

Overall, this is a strong and valuable contribution. Addressing the points above would further strengthen its theoretical clarity and methodological transparency.

Reviewer #2: I would like to thank the authors for this interesting manuscript. The paper addresses an important topic and has the potential to make a useful contribution to the literature on migrant health, life expectancy, and disability-free life expectancy. The use of detailed country-of-birth data across three time points is a clear strength. However, I believe that several major issues need to be addressed before the manuscript can be considered for publication.

1. Title and focus of the paper

The full title suggests that the paper focuses primarily on spatial inequalities in life expectancy and disability-free life expectancy in England and Wales. However, the short title and, more importantly, the manuscript itself focus mainly on inequalities by country of birth / immigrant background. I would therefore recommend revising the title so that it more accurately reflects the actual content and emphasis of the paper.

2. Abstract

The abstract would benefit from substantial revision in terms of flow, clarity, and readability. At present, several terms are not sufficiently clear. For example, it is unclear what is meant by “total population mortality” and “lowest-level country of origin”. It would also be helpful to state how many country-of-birth groups are included in the analysis.

The description of the data should also be clarified. The abstract states that the study uses “total population mortality and census data from 2000–02, 2010–12 and 2020–22”. However, census data refer to a single point in time, whereas mortality data appear to be aggregated over three-year periods around each census. This distinction should be made explicit.

I would also suggest removing the statement “providing the most granular analysis of life expectancy (LE) and disability-free life expectancy (DFLE) in the global evidence base to date” from the middle of the abstract. Instead, the abstract should more clearly state that DFLE is estimated using self-reported limiting long-term illness.

In addition, the term “inequalities” should be defined more precisely. If this refers to absolute or relative differences in LE and DFLE between each immigrant group and the England and Wales-born population, this should be stated explicitly. Finally, the repeated cross-sectional / longitudinal aspect of the study is an important strength, but it is not sufficiently highlighted in the abstract.

3. Data sources, missing values, and numerator–denominator bias

The manuscript should explain more clearly how missing values were handled, particularly for country of birth in death registrations and census data, and for health status in the census.

I would suggest discussing whether census undercounting, especially among some migrant groups, may affect the results. Since deaths and population exposures by country of birth come from different sources, there is a potential risk of numerator–denominator bias. This issue deserves more explicit discussion.

4. Small numbers and smoothing at older ages when constructing LT

Have you considered applying any smoothing or correction methods, particularly at older ages? Or was instability at older ages not a substantial issue in these datasets?

5. Description of the Sullivan method.

The statement “From the values for remaining years of life expectancy we use the Sullivan method to calculate the expected number of years being disability-free given the prevalence of disability” is not sufficiently clear. Please provide a more precise description of how the Sullivan method was implemented. In addition, did you estimate 95% confidence intervals for DFLE? If so, this should be stated explicitly in the Methods section, together with the relevant references.

6. The size of inequalities

Much of the analysis is based on comparisons between immigrant groups and the England and Wales-born population. I would therefore recommend presenting the numeric estimates of the gaps in LE and DFLE, together with corresponding confidence intervals. This would make the comparisons more transparent and help readers assess whether observed differences are meaningful.

7. Classification of groups

The classification of groups into HIE, MMP, UIE, and SLBH is useful, but the criteria should be made clearer. In particular, the manuscript states that when differences are marginal or not statistically significant, groups are classified into the “most appropriate category”. This is potentially subjective. I would recommend the authors to explain exactly how these decisions were made and whether classifications are based on statistical significance, point estimates or substantive thresholds.

8. Supplementary analyses

The supplementary analyses are extensive, but their role in the manuscript is not always clear. Some appear to be sensitivity analyses, while others provide descriptive contextual information. I would recommend distinguishing more clearly between supplementary analyses and sensitivity analyses and retaining in the main text only those results that directly support the conclusions.

**********

what does this mean?). If published, this will include your full peer review and any attached files.

If you choose “no”, your identity will remain anonymous but your review may still be made public.

**Do you want your identity to be public for this peer review?**  For information about this choice, including consent withdrawal, please see our Privacy Policy

Reviewer #1: No

Reviewer #2: No

**********

[NOTE: If reviewer comments were submitted as an attachment file, they will be attached to this email and accessible via the submission site. Please log into your account, locate the manuscript record, and check for the action link "View Attachments". If this link does not appear, there are no attachment files.]

To ensure your figures meet our technical requirements, please review our figure guidelines: https://journals.plos.org/plosone/s/figures

You may also use PLOS’s free figure tool, NAAS, to help you prepare publication quality figures: https://journals.plos.org/plosone/s/figures#loc-tools-for-figure-preparation.

NAAS will assess whether your figures meet our technical requirements by comparing each figure against our figure specifications.

# Author comment: Author response to Decision Letter 1

**DOI:** 10.1371/journal.pone.0356481.r002

24 Jun 2026

Please see response document that we have uplaoded

Attachment Submitted filename: response_letter_fin.docx

# Peer review: Decision Letter 1

**DOI:** 10.1371/journal.pone.0356481.r003

**Authors:** Eyal Bar-Haim

4 Aug 2026

Immigrant life expectancy and disability free life expectancy by detailed country of birth in England and Wales: a total population, repeated cross-sectional approach

PONE-D-25-65095R1

Dear Dr. Harrison,

We’re pleased to inform you that your manuscript has been judged scientifically suitable for publication and will be formally accepted for publication once it meets all outstanding technical requirements.

Within one week, you’ll receive an e-mail detailing the required amendments. When these have been addressed, you’ll receive a formal acceptance letter and your manuscript will be scheduled for publication.

An invoice will be generated when your article is formally accepted. Please note, if your institution has a publishing partnership with PLOS and your article meets the relevant criteria, all or part of your publication costs will be covered. Please make sure your user information is up-to-date by logging into Editorial Manager at Editorial Manager® and clicking the ‘Update My Information' link at the top of the page. For questions related to billing, please contact billing support.

If your institution or institutions have a press office, please notify them about your upcoming paper to help maximize its impact. If they’ll be preparing press materials, please inform our press team as soon as possible -- no later than 48 hours after receiving the formal acceptance. Your manuscript will remain under strict press embargo until 2 pm Eastern Time on the date of publication. For more information, please contact onepress@plos.org.

Kind regards,

Eyal Bar-Haim

Academic Editor

PLOS One

Additional Editor Comments (optional):

Reviewers' comments:

Reviewer's Responses to Questions

**Comments to the Author**

Reviewer #1: All comments have been addressed

**********

2. Is the manuscript technically sound, and do the data support the conclusions?

Reviewer #1: Yes

**********

3. Has the statistical analysis been performed appropriately and rigorously?

Reviewer #1: Yes

**********

4. Have the authors made all data underlying the findings in their manuscript fully available?

The PLOS Data policy

Reviewer #1: Yes

**********

5. Is the manuscript presented in an intelligible fashion and written in standard English?

Reviewer #1: Yes

**********

Reviewer #1: The authors have successfully addressed all of my comments. I look forward to seeing the article in print.

**********

what does this mean?). If published, this will include your full peer review and any attached files.

If you choose “no”, your identity will remain anonymous but your review may still be made public.

**Do you want your identity to be public for this peer review?**  For information about this choice, including consent withdrawal, please see our Privacy Policy

Reviewer #1: No

**********

# Editor report: Acceptance letter

**DOI:** 10.1371/journal.pone.0356481.r004

**Authors:** Eyal Bar-Haim

PONE-D-25-65095R1

PLOS One

Dear Dr. Harrison,

I'm pleased to inform you that your manuscript has been deemed suitable for publication in PLOS One. Congratulations! Your manuscript is now being handed over to our production team.

At this stage, our production department will prepare your paper for publication. This includes ensuring the following:

* All references, tables, and figures are properly cited

* All relevant supporting information is included in the manuscript submission,

* There are no issues that prevent the paper from being properly typeset

You will receive further instructions from the production team, including instructions on how to review your proof when it is ready. Please keep in mind that we are working through a large volume of accepted articles, so please give us a few days to review your paper and let you know the next and final steps.

Lastly, if your institution or institutions have a press office, please let them know about your upcoming paper now to help maximize its impact. If they'll be preparing press materials, please inform our press team within the next 48 hours. Your manuscript will remain under strict press embargo until 2 pm Eastern Time on the date of publication. For more information, please contact onepress@plos.org.

You will receive an invoice from PLOS for your publication fee after your manuscript has reached the completed accept phase. If you receive an email requesting payment before acceptance or for any other service, this may be a phishing scheme. Learn how to identify phishing emails and protect your accounts at https://explore.plos.org/phishing.

If we can help with anything else, please email us at customercare@plos.org.

Thank you for submitting your work to PLOS ONE and supporting open access.

Kind regards,

PLOS ONE Editorial Office Staff

on behalf of

Dr. Eyal Bar-Haim

Academic Editor

PLOS One
